# Supplementary material for: Biomolecular interactions modulate macromolecular structure and dynamics in atomistic model of a bacterial cytoplasm
Source: eLife. 2016 Nov 1;5:e19274. doi: 10.7554/eLife.19274 (PMC5089862; doi:10.7554/eLife.19274)
Supplement: Supplementary file 1. — List of Macromolecules. Copy numbers for each macromolecule (represented by tag name) in four simulation systems. The Stokes radius Rs is given in the last column. Groups and types of metabolites. Net charge and number of copies for each metabolite (represented by tag name) in three simulation systems. Phosphates are highlighted with a pink background. DOI: http://dx.doi.org/10.7554/eLife.19274.027 [file elife-19274-supp1.docx]

**List of Macromolecules.** Copy numbers for each macromolecule (represented by tag name) in four simulation systems. The Stokes radius *R*_s_ is given in the last column.

**Groups and types of metabolites.** Net charge and number of copies for each metabolite (represented by tag name) in three simulation systems. Phosphates are highlighted with a pink background.
